# A Case of Severe Teratozoospermia and Infertility Due to Homozygous Mutation c.144delC in the AURKC Gene

**DOI:** 10.7759/cureus.43376

**Published:** 2023-08-12

**Authors:** Meriame Abbassi, Hanane Sayel, Hinde El Mouhi, Meryem Jelte, Mohamed Ahakoud

**Affiliations:** 1 Laboratory of Biomedical and Translational Research, Faculty of Medicine and Pharmacy and Dental Medicine, Sidi Mohammed Ben Abdellah University, Fez, MAR; 2 Medical Genetics and Oncogenetics Laboratory, Central Laboratory of Medical Analysis, University Hospital Center Hassan II, Fez, MAR; 3 Center for Doctoral Studies Engineering Sciences and Techniques, Faculty of Sciences and Technologies, Sidi Mohammed Ben Abdellah University, Fez, MAR; 4 Laboratory of Biotechnology, Environment, Agri-Food and Health, Faculty of Sciences Dhar El Mahraz, Sidi Mohamed Ben Abdellah University, Fez, MAR

**Keywords:** sexual health, macrozoospermia, severe teratozoospermia, male infertility, c.144delc mutation, aurkc gene

## Abstract

This case report focuses on a 33-year-old male patient with a history of infertility, characterized by severe micro-oligo-asthenospermia. Subsequent analysis revealed the presence of multi-headed and multi-flagellated spermatozoa, indicating a potential case of macrocephalic spermatozoa syndrome linked to a mutation in the *AURKC* gene. Genetic testing confirmed the presence of a pathogenic mutation, c.144delC, in a homozygous state in the *AURKC* gene. The *AURKC *gene is known to play a vital role in meiosis during sperm production, and its mutation can lead to abnormalities in sperm morphology and function, resulting in conditions like macrozoospermia and male infertility. Additionally, the patient was diagnosed with a grade III varicocele on the left testicle, which further contributed to his infertility. Varicoceles are associated with decreased sperm production and quality, making them one of the common reversible causes of male infertility. This case highlights the significance of comprehensive diagnostic approaches, including spermogram, ultrasonography, and genetic testing, in managing male infertility cases. It also emphasizes the intricate interplay between genetic mutations and physical conditions in the manifestation of male infertility. Further research is warranted to elucidate the mechanisms underlying AURKC-related sperm abnormalities and to develop effective therapeutic interventions. Moreover, a deeper understanding of such genetic factors may aid in the development of genetic counseling strategies for couples experiencing infertility.

## Introduction

Male infertility is a multifaceted problem that can be attributed to a variety of factors. It has been estimated to affect approximately 7% of men worldwide, playing a role in around half of all cases of couple infertility [1]. Male infertility is usually characterized by the decreased production and quality of sperm [2]. Severe cases can exhibit conditions like oligospermia or low sperm count, asthenospermia or reduced mobility of sperm, and teratospermia or abnormal sperm morphology [3]. An intriguing phenotype in this realm is macrozoospermia, where the spermatozoa have abnormally large heads [4].

This rare syndrome is most commonly caused by mutations in the Aurora kinase C (*AURKC*) gene [5]. The *AURKC* gene encodes a member of a highly conserved family of serine/threonine kinases, which play a pivotal role in chromosomal segregation during mitosis and meiosis [5]. Aberrant expression or function of this protein due to pathogenic mutations can disrupt the proper formation of the mitotic spindle, consequently leading to cytokinesis failure and resulting in macrozoospermia [5].

This condition often presents with a high degree of spermatozoa head anomalies, including multi-tailed and multi-headed forms [4]. The current report presents an intriguing case of a 33-year-old male presenting with infertility. The patient’s spermogram revealed severe micro-oligo-asthenospermia, and further investigation revealed the presence of multi-headed and multi-flagellated spermatozoa, strongly suggesting a case of macrozoospermia [4].

Adding to the complexity of the patient’s condition was the finding of a grade III varicocele on the left testicle. Varicocele, an abnormal dilation of the veins in the pampiniform plexus within the scrotum, is a well-established cause of male infertility [6]. It accounts for approximately 40% of all cases of primary infertility and 80% of secondary infertility [6]. The exact mechanism by which varicoceles contribute to infertility is not entirely understood, but it's generally accepted that they raise the temperature of the testes, impairing spermatogenesis, and causing a decrease in sperm count, motility, and morphology [6].

The presence of both a potential genetic factor (*AURKC* mutation) and a physical factor (varicocele) adds layers of complexity to the patient’s condition [7]. As such, the case underlines the significance of an integrated approach in diagnosing and managing male infertility, underscoring the need for the involvement of genetic analysis, ultrasonography, and standard semen analysis [7]. This case serves as a stark reminder of the intricate network of genetic and physical factors contributing to male infertility [8].

More research is urgently needed to comprehend the underpinnings of these complex interactions. A better understanding of the impact of *AURKC* mutations on spermatozoa morphology, and the implications of conditions such as varicoceles on sperm production and quality, may pave the way for improved therapeutic strategies and genetic counseling for affected couples [7].

## Case presentation

A 33-year-old male presented at our fertility clinic after a prolonged period of unsuccessful conception attempts spanning two years. He had an unremarkable medical history, with no traces of sexually transmitted diseases, substance abuse, exposure to radiation, or past chemotherapy. Physical examination identified a left-sided grade III varicocele.

The patient's semen analysis unveiled the presence of severe oligozoospermia, identified by a sperm concentration of 1 million/mL, markedly below the normative value of >15 million/mL. Asthenospermia was also noted with only 20% progressive motility, in contrast to the standard >32%. Furthermore, teratospermia was established, with a strikingly low percentage, less than 4%, of spermatozoa exhibiting normal morphology (normal range: >4%). Microscopically, a significantly elevated frequency of multi-headed and multi-flagellated spermatozoa was observed, indicative of potential macrozoospermia. This observation gave rise to the assumption that there may exist an underlying genetic aberration, thereby warranting a targeted genetic analysis.

Genetic investigation utilized Sanger sequencing, employing the polymerase chain reaction (PCR) amplification of the target region, which was exon 3 of *AURKC* gene. Subsequent purification and sequencing of the PCR products were achieved using the BigDye™ Terminator v3.1 Cycle Sequencing Kit (Thermo Fisher Scientific Inc., Waltham, Massachusetts, United States). The sequencing reactions underwent purification, followed by an analysis using the ABI PRISM® 3100 Genetic Analyzer (Thermo Fisher Scientific, Inc.). The resulting data were interpreted using the sequencing analysis software, Sequence Scanner v1.0 (Thermo Fisher Scientific Inc.).

**Figure 1:**
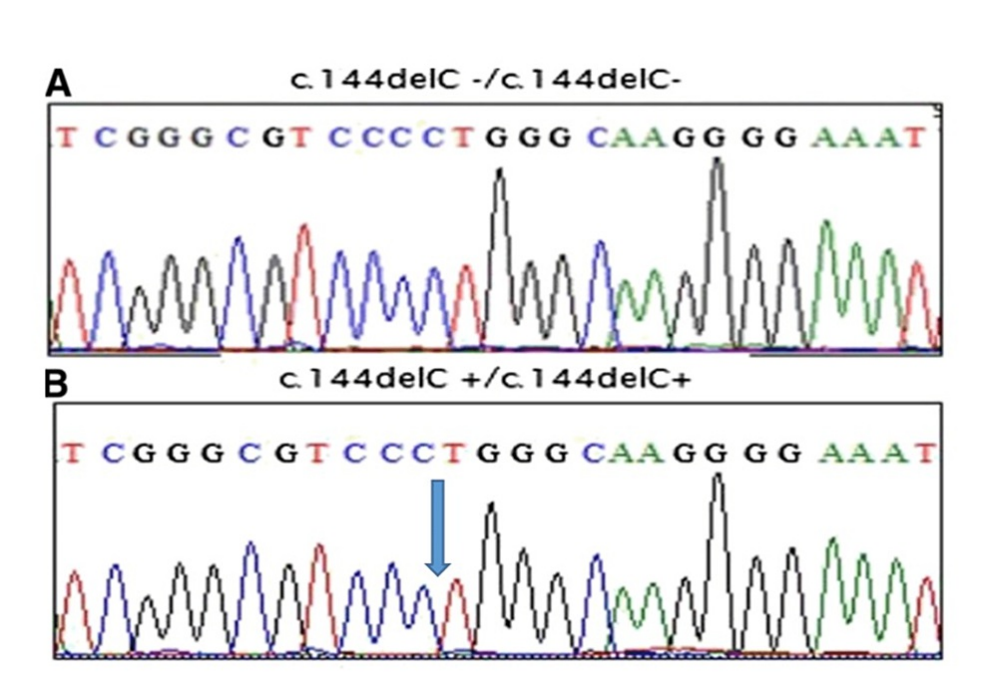
Sequence chromatograms showing the c.144delC mutation. (A) Wild type; (B) homozygous deletion (blue arrow)

Through a meticulous examination of the sequencing data, a pathogenic homozygous mutation was discovered, designated c.144delC, within exon 3 of the *AURKC* gene in the patient's DNA (Figure 1). This particular mutation has previously been documented in association with macrozoospermia, thus reinforcing our initial assumption based on the patient's sperm morphology anomalies.

Table 1 summarizes the clinical and genetic features of the patient, providing a comprehensive overview of the observed phenotype and the identified genetic alteration.

**Table 1:** Clinical and genetic features N/A not applicable Note: The normal range values are based on standard reference values for adult males. Hormone levels may vary slightly depending on the laboratory's reference range. FSH: follicle-stimulating hormone; LH: luteinizing hormone

Category	Description	Normal Range
Age at diagnosis	33 years	N/A
Medical history	No significant medical history	N/A
Family history	No known family history of reproductive disorders	N/A
Physical examination	Normal male genitalia, secondary sexual characteristics present	N/A
Semen analysis	Macrozoospermia; 5 million sperm/mL (oligozoospermia)	≥ 15 million sperm/mL
	50% progressive motility (asthenozoospermia)	≥ 32% progressive motility
	100% large-headed sperm (teratozoospermia)	≤ 96% normal-shaped sperm
Hormone levels	Normal FSH, LH, and testosterone	FSH: 3-15 mIU/mL; LH: 1-9 mIU/mL; testosterone: 300-1,000 ng/dL
Karyotype	Normal male karyotype, 46, XY	N/A
Molecular genetic testing	Homozygous pathogenic mutation in the *AURKC *gene (c.144delC)	N/A

This mutation, c.144delC, is a deletion mutation that results in a frameshift, leading to a subsequent premature stop codon. Consequently, this alteration disrupts the translation of the *AURKC* gene, producing an abnormally short and dysfunctional AURKC protein. The impairment of this protein directly impacts the completion of spermiogenesis, culminating in macrozoospermia.

The identification of this homozygous mutation c.144delC within the *AURKC* gene establishes a clear link to the observed severe oligo-astheno-teratozoospermia and macrozoospermia in our patient. Our findings, therefore, underline the criticality of incorporating advanced genetic diagnostics like Sanger sequencing in infertility clinics. This approach aids in the diagnosis of complex conditions such as male infertility and provides key insights for genetic counseling while shedding light on potential therapeutic strategies.

## Discussion

The observed clinical phenotype of severe oligo-astheno-teratozoospermia and macrozoospermia, and the subsequent identification of a homozygous pathogenic mutation (c.144delC) in the *AURKC *gene in our male patient significantly contribute to the expanding literature on the genetic basis of male infertility. AURKC has been previously identified as a vital player in spermatogenesis, particularly in the late stage of spermiogenesis, thereby establishing a plausible genetic link between mutations in this gene and abnormal sperm morphology, such as macrozoospermia [5,9].

AURKC, located on chromosome 19q13.43, encodes a member of the Aurora subfamily of serine/threonine protein kinases that are involved in the regulation of chromosomal segregation and cytokinesis [10]. Several studies have suggested that AURKC has a unique role during the meiosis stage of germ cell maturation, especially during chromosomal segregation [11,12]. As such, mutations in the *AURKC* gene have the potential to disrupt this critical process, leading to abnormal sperm formation and, consequently, male infertility.

The c.144delC mutation identified in our patient is a deletion mutation causing a frameshift that results in a premature stop codon. This, in turn, leads to the production of a truncated AURKC protein. Previous reports have linked the presence of this specific mutation to macrozoospermia [4,13]. Our findings corroborate these studies and further solidify the association between the c.144delC mutation and macrozoospermia.

Moreover, previous reports have documented that macrozoospermia is often associated with a higher prevalence of chromosomal aneuploidies in spermatozoa, including both sex and autosomal chromosomes [14]. This can potentially increase the risk of adverse pregnancy outcomes, including miscarriages and birth defects, should fertilization occur [15]. Thus, the presence of this mutation in infertile men, particularly those exhibiting macrozoospermia, necessitates robust genetic counseling and potentially, the consideration of advanced reproductive techniques such as intracytoplasmic sperm injection (ICSI) combined with preimplantation genetic testing for aneuploidies (PGT-A) [16].

Our findings also underscore the pivotal role of genetic testing in the diagnostic workflow for male infertility. Despite the high prevalence of male factor infertility, only a fraction of cases are attributed to identifiable genetic causes, highlighting the complexity of this condition and the need for more comprehensive diagnostic approaches [17]. As such, the incorporation of techniques such as Sanger sequencing into clinical practice can be a valuable tool for identifying underlying genetic causes, thereby aiding in diagnosis, prognosis, and personalized treatment strategies.

However, while our study adds to the body of knowledge about AURKC's role in male infertility, more research is needed. Larger cohorts of infertile men need to be screened for mutations in *AURKC* and other genes implicated in male infertility, to better understand the spectrum of genetic causes. Furthermore, functional studies are needed to clarify the exact mechanisms by which these mutations disrupt spermatogenesis and contribute to infertility.

## Conclusions

This report highlights the significance of *AURKC* mutations in male infertility and supports the routine inclusion of genetic testing in the diagnostic evaluation of male infertility, particularly in cases with severe sperm morphology anomalies. There is a substantial role of genetic factors, particularly the homozygous pathogenic mutation c.144delC in the *AURKC* gene, in the development of severe oligo-astheno-teratozoospermia and macrozoospermia in an infertile male patient. The identification of this mutation adds to the growing body of evidence linking *AURKC* mutations to abnormal sperm morphology and male infertility. Incorporating genetic testing, such as Sanger sequencing, into the diagnostic workup for male infertility is essential. Identifying specific genetic mutations provides crucial insights into the mechanisms underlying impaired spermatogenesis, enabling personalized treatment strategies and genetic counseling.

Furthermore, the association between the c.144delC mutation in *AURKC* and macrozoospermia calls for further research to elucidate AURKC's exact role in spermiogenesis and its impact on male fertility. Large-scale cohort studies involving diverse populations will help establish the prevalence of *AURKC* mutations and their global association with male infertility. With the advancements in genetic testing technologies, including next-generation sequencing, a more comprehensive understanding of the genetic basis of male infertility is within reach. This knowledge holds promise for the development of novel diagnostic tools, targeted therapies, and family planning strategies for affected individuals. This report thus contributes to the advancement of knowledge in the field of reproductive medicine.
